# Psychometric Properties and Normative Data Using Item Response Theory Approach for Three Neuropsychological Tests in Waranka Children Population

**DOI:** 10.3390/healthcare13040423

**Published:** 2025-02-15

**Authors:** María José Fierro Bósquez, Eliana María Fuentes Mendoza, Laiene Olabarrieta-Landa, Trinidad Abiuso Lillo, Erick Orozco-Acosta, Guido Mascialino, Juan Carlos Arango-Lasprilla, Diego Rivera

**Affiliations:** 1 Department of Health Science, Public University of Navarre, 31006 Pamplona, Spain; fierro.151061@e.unavarra.es (M.J.F.B.); fuentes.151056@e.unavarra.es (E.M.F.M.); laiene.olabarrieta@unavarra.es (L.O.-L.); abiuso.166440@e.unavarra.es (T.A.L.); diego.rivera@unavarra.es (D.R.); 2 Instituto de Investigación Sanitaria de Navarra (IdiSNA), 31008 Pamplona, Spain; 3 Engineering Faculty, Universidad Simón Bolívar, Barranquilla 080002, Colombia; erick.orozco@unisimon.edu.co; 4 Life Sciences Research Center, Universidad Simón Bolívar, Barranquilla 080002, Colombia; 5 Escuela de Psicología y Educación, Universidad de Las Américas, Quito 170411, Ecuador; guido.mascialino@udla.edu.ec; 6 Department of Psychology, Virginia Commonwealth University, Richmond, VA 23284, USA

**Keywords:** normative data, children, item response models, indigenous population, regression models

## Abstract

Objective: To evaluate the psychometric properties of the Shortened Version of the Token Test (SVTT), the Peabody Picture Vocabulary Test (PPVT-III), and the Rey–Osterrieth Complex Figure (ROCF) using an item response theory (IRT) framework and to establish normative data for Waranka children and adolescents based on their ability scores. Methods: A total of 436 healthy people aged between 6 and 17 participated in this study. The factor structure was evaluated using confirmatory factor analysis (CFA) and the difficulty and discrimination parameters using IRT through the two-parameter logistic model for the SVTT and PPTV-III, while for the ROCF, the graded response model was used. The ordinal alpha and McDonald’s omega were used for reliability. Results: For most items, a low ability was enough to achieve the highest scores for the ROCF and SVTT. For the PPVT-III, the items aligned sequentially based on the difficulty, and a low level of ability was enough to achieve the highest score for the first 40 items. The ROCF, SVTT, and PPVT-III demonstrated adequate reliability. The ROCF copy and immediate recall scores were influenced by the mean parents’ years of education (MPE) and quadratic age interaction. The SVTT performance was affected by the quadratic age and sex interaction, and the PPVT-III by the interaction effect of the MPE and quadratic age. Conclusions: This is the first study to analyze the psychometric properties of the ROCF, SVTT, and PPVT-III through IRT models that are accurate and relevant for the validity of the obtained data and generate normative data in the under-represented nation of Ecuador for clinical and research purposes.

## 1. Introduction

Ecuador is an intercultural nation composed by 18 under-represented ethnic populations (UEPs) [1]. Among these, the Waranka people are an indigenous population residing in rural areas of the Sierra region, specifically in Guaranda, at altitudes ranging from 2500 to 3500 m above sea level [2]. The Waranka community preserves its unique cultural, social, political, and economic characteristics, as well as its ancestral language, Kichwa [3]. This cultural and linguistic diversity suggests that health services, including neuropsychological care, must be adapted to meet the specific needs of this population.

UEPs are at higher risk of developing health issues, including several disorders and diseases associated with brain damage [4] and cognitive difficulties [5,6]. As such, neuropsychological services become essential to appropriately face these health issues. Indeed, neuropsychological assessment allows for obtaining an accurate profile of the patient, providing insight into potential cognitive deficits. This assessment is based on observation, interview and the examination of various cognitive processes (i.e., perception, attention, memory), followed by neuropsychological analysis of the data obtained [7]. Neuropsychological tests need to have satisfactory psychometric properties to obtain adequate results from the evaluation. These properties are validity, reliability, sensitivity, specificity, measurement errors and normative data (also known as references values or norms) [8].

In Latin America, a significant challenge in neuropsychological practice is the lack of locally relevant normative data for cognitive tests. Normative data are essential for interpreting test results, as they allow clinicians to compare an individual’s performance to that of a reference group with similar sociodemographic characteristics [9,10]. Without such norms, neuropsychologists face difficulties in making accurate diagnoses and intervention decisions [11]. A national survey in Ecuador identified this limitation as one of the primary challenges for practitioners in the field, despite the widespread use of tests such as the ROCFT, the SVTT, and the Boston Naming Test [12]. Efforts to address this issue have included studies providing normative data for adult populations in Quito [13] and for children across Latin America, including Ecuador [14].

However, even when normative data are available, they may not adequately represent indigenous populations such as the Waranka. UEPs often exhibit distinct sociocultural and linguistic characteristics that differ significantly from those of urban or majority populations. This raises concerns about the applicability of existing norms and the validity of commonly used neuropsychological tests for these communities. In such cases, advanced statistical methods are essential to evaluate whether these tests are reliable and valid for indigenous populations.

Item response theory (IRT) offers a robust framework for examining the psychometric properties of neuropsychological tests. Unlike classical test theory (CTT), IRT allows for the evaluation of both individual items and the overall test, modeling the probability of an individual’s response based on their ability and the item characteristics [15,16]. This approach provides several advantages, including the estimation of invariant parameters and the ability to assess the goodness of fit of different models to the data [17]. By leveraging IRT, it is possible to determine whether tests like the SVTT, PPVT, and ROCF are appropriate for use in indigenous populations and to develop ability-based norms that better reflect the unique characteristics of these groups.

Given these considerations, the objectives of this study were twofold: (1) to evaluate the psychometric properties (specifically, the construct validity, reliability, internal consistency and discrimination and difficulty parameters) of the SVTT, PPVT, and ROCF, and (2) to establish ability-based normative data for Waranka children and adolescents

## 2. Materials and Methods

### 2.1. Participants

The sample consisted of 436 children and adolescents belonging to the Waranka community in Ecuador. Most of the sample (55.64%) were female, with an average age of 11.52 (SD = 3.19) and mean years of parents’ education (MPE) of 7.89 (SD = 3.58). For the estimation of the sample size, the optimal sample size in the regression approach from Innoncenti et al. [10] was used. The equation is comprised of the following elements: confidence interval value = 95%; number of predictors in the final model = 4; percentile value to be considered “abnormal” = −1.65 (percentile 5), and desired margin of error estimation = 0.24. Regarding the sample, nonproportionate quota sampling was used, looking for a symmetrical distribution for the strata of age and sex.

To be eligible for study participation, all the participants had to meet the following inclusion criteria: (a) to be between 6 and 17 years old, (b) to have been born and live in the Waranka indigenous community, (c) to have an IQ ≥ 80 on the Test of Nonverbal Intelligence [18], and (d) to have a score of <19 on the Children’s Depression Inventory [19]. Participants were excluded if they reported any of the following: (a) learning problems, (b) a history of psychoactive substance use (in adolescents’ case), and (c) a score < 5 for the Alcohol Use Disorder Identification Test (AUDIT-C) [20].

Nineteen participants were excluded due to the inclusion/exclusion criteria. Also, 18 participants were excluded because they did not complete the SVTT and the PPVT-III. As a result, the final sample was composed of 399 participants.

### 2.2. Instruments

#### 2.2.1. Rey–Osterrieth Complex Figure (ROCF)

This test evaluates perception in visual–spatial construction and visual immediate recall. For this study, Figure S1 was used, which includes 18 items. In the first part of the test, the examinee is prompted to copy a figure. Once finished, the figure is removed, and after three minutes of delay, the examinee is asked to reproduce what s/he remembers about the figure. For scoring, the following criteria are considered: 2 points are given for accurate reproduction of the element, and 1 point is awarded for reproductions that are distorted, incomplete but properly placed, or completed but poorly positioned. Additionally, 0.5 points are granted if the element is both distorted or incomplete and poorly placed, and a score of 0 is assigned when the element is absent or unrecognizable [21].

#### 2.2.2. Shortened Version of the Token Test (SVTT)

The SVTT aims to evaluate verbal comprehension through the fulfillment of commands. For the task, 20 tokens of different sizes, colors and shapes are used. The examinee is asked to follow certain commands, with an increasing level of difficulty, that require the use of tokens. This test is divided into 6 parts, and in the scoring system, 1 point is awarded for each correct response, while 0.5 points are granted if the correct response is given after receiving instructions for the second time [22]. However, if the participant still fails to respond correctly after repetition of the instructions, s/he will receive a score of 0.

#### 2.2.3. Peabody Picture Vocabulary Test-III (PPVT-III)

The PPVT-III is designed to assess receptive vocabulary and serves as a quick screening tool to detect potential issues in verbal ability. It consists of 192 items arranged in increasing difficulty. For each item, black and white pictures are presented, and the examinee must choose the image that best represents the meaning of a word provided verbally. Each item is scored dichotomously as 0 if the response is incorrect or 1 if it is correct [23].

### 2.3. Procedure

The participants completed a neuropsychological battery that included the PPVT-III, SVTT, and ROCF. To minimize bias and ensure consistency, the order of the test administration was randomized for each participant, and a Microsoft Excel template was used to reduce potential errors in the data entry. This study received approval from the Ethics Committee of the Public University of Navarre and adhered to the principles outlined in the Declaration of Helsinki.

The research team from the Public University of Navarre provided training to the Ecuadorian data collection team, led by M.J., on the proper administration and scoring of the tests. For recruitment, the team initially met with community leaders to present and explain the project. Once the leaders approved, they facilitated meetings with parents in the community, during which the researchers explained this study in detail and addressed any questions. Parents or guardians who agreed to participate signed an informed consent form and completed a questionnaire collecting sociodemographic information, medical history, and health status of their child. The tests were administered individually in intercultural bilingual schools, with each session lasting between 90 and 120 min. Data collection occurred from July 2022 to December 2022. Participation in this study was voluntary, and no financial compensation was provided.

### 2.4. Statistical Analysis

#### 2.4.1. Psychometric Properties

To evaluate the construct validity of the tests, confirmatory factor analyses (CFAs) were performed using the diagonally weighted least squares (DWLS) estimator to test the unidimensionality in each test. The model fit was evaluated using the following indices: the ratio of the chi-square (χ^2^) statistic to the degrees of freedom (χ^2^/df), with acceptable values being less than or equal to 2; the Comparative Fit Index (CFI) and Tucker–Lewis Index (TLI), with values of 0.95 or higher being considered indicative of an excellent model fit; the root mean square error of approximation (RMSEA), with thresholds of 0.06 for an excellent fit reported, along with its 90% confidence interval (CI_90%_); and the standardized root mean square residual (SRMR), with values below 0.08 deemed acceptable [24].

To assess the reliability of the ROCF, SVTT, and PPVT-III, both the ordinal alpha (α) and McDonald’s omega (ɷ) coefficients [25] were employed. McDonald’s omega addresses the limitations of α by providing a measure that represents the ratio of the true score variance, calculated from the model’s estimated parameters, to the total variance of the items, including their variances and covariances as represented by the model [26].

IRT was used to obtain the difficulty and discrimination parameters through the two-parameter logistic (2PL) model for the SVTT and PPTV-III dichotomous items. This model scores individuals and shows which items have mismatches, and it is less restrictive than the Rasch model [27]. For the polytomous items of the ROCF copy and ROCF immediate recall, a graded response model (GRM) was used, since it estimates both the likelihood of selecting a specific response to an item and the extent to which an item effectively measures respondents’ underlying trait [28]. In this study, the ability score (*θ*) derived from the IRT models was utilized as a key metric to represent the underlying trait levels of individuals. This score reflects the probability of an individual responding correctly (or at a specific level for polytomous items) based on the interaction between their latent trait and the item parameters, such as the difficulty (*b*) and discrimination (*a*). Unlike raw scores, the ability score provides a more precise and interpretable measure of individual performance, accounting for the characteristics of the test items and allowing for meaningful comparisons across individuals and items on a standardized scale.

#### 2.4.2. Demographic Effects

The effects of the demographic variables on the ROCF copy, ROCF immediate recall, SVTT, and PPVT-III performance were analyzed using multiple linear regression based on the ability (*θ*) in each test [29]. These final regression model included Age, Age^2^, *In*(MPE), sex, and all the possible two-way interactions among these variables as predictors. Age^2^ was obtained using a procedure to compute orthogonal polynomials, warranting the independence assumption between covariates. MPE was depicted on a natural logarithmic scale, since we anticipated an increase in the test performance with the initial years of education, followed by a gradual deceleration in growth. The full regression model had this equation:

$$\theta_{i}=\beta_{0}+\beta_{1}{\left(Age\right)}_{i}+\beta_{2}\;{\left(Age\right)}_{i}^{2}+\beta_{3\;}\left[\ln{\left(MPE\right)}_{i}\right]+\beta_{4}\;{\left(sex\right)}_{i}+\cdots +\beta_{k}\;{Interactions}_{i}+\varepsilon_{i}.$$

The model postulates that the residuals *ε_i_* follow a normal distribution with a mean of 0 and variance $\sigma_{e}^{2}$ that is $\varepsilon_{i}\;\sim \;N(0,\;\sigma_{e}^{2})$. For the variable selection, the exhaustive subset selection approach was used. This exhaustive approach evaluates all the possible combinations of *p* variables, amounting to $2^{p}$ models—for instance, in our case, 2^9^ = 512 models—to identify the best-fitting set for a model by systematically generating and comparing the performance of all the models and selecting the optimal one based on the Bayesian information criterion (BIC). To further validate the robustness of the model and assess the potential overfitting, we applied a 5-fold cross-validation approach, which partitions the data into five subsets, iteratively training the model on four subsets and testing it on the remaining one, ensuring a balanced evaluation of predictive performance across folds [30].

For the final multiple linear regression models, the following assumptions were assessed: (a) the multicollinearity was computed by the variance inflation factor (VIF) (≤10), (b) concerning homoscedasticity, the Levene test was employed, (c) the normality of the standardized residuals was evaluated employing the Kolmogorov–Smirnov test, and (d) the influential values were determined by applying the maximum Cook’s distance *D_i_*, which should not exceed 1 [31].

### 2.5. Normative Data Procedure

Van Der Elst et al.’s [32] steps were followed to generate normative conversions of the *θ_s_* sores for the ROCF copy and immediate recall, SVTT and PPVT-III into demographically corrected percentile values. For simplicity, ${\hat{\theta }}_{i}$ and ${\hat{\beta }}_{k}$ are estimates of *θ_i_* and *β_k_*, respectively. First, the expected ability scores were computed by means of the final regression model ${\hat{\theta }}_{i}={\hat{\beta }}_{0}+{\hat{\beta }}_{1}X_{1}+\cdots +{\hat{\beta }}_{k}X_{k}.$ Second, the residuals were calculated (*e_i_* = observed ability score—expected ability score). Third, the residuals were standardized *z_i_* = *e_i_*/*SD* (residual), with *SD* (residual) = the standard deviation of the residuals in the normative sample. Fourth, by employing the standard normal distribution function, we ascertained the precise percentile value corresponding to the previously calculated *z_*i*_*, following confirmation of the normal distribution of the data. All the analyses were conducted through R Project for Statistical Computing for Windows [33] using the following libraries: *lavaan* package [34], *mIRT* package [35] and *ltm* package [36].

## 3. Results

### 3.1. Psychometric Properties

#### 3.1.1. ROCF Copy and Immediate Recall

For the ROCF copy, CFA supports the assumption of unidimensionality (χ^2^/df = 1.722; CFI = 0.996; TLI = 0.995; RMSEA = 0.043 [0.033, 0.052]; SRMR = 0.051). In addition, the ROCF copy’s ordinal alpha was α = 0.95 and the McDonald’s omega was ɷ = 0.94.

The item parameters generated through the IRT analyses for the ROCF copy (see Table 1) showed that the discrimination values ranged from 1.32 (item 7: small segment) to 2.59 (item 11: circle with three dots). For instance, an individual obtaining a latent value, as seen in the boundary 1 column, where Item 1 = −2.168, has a 50% probability of achieving a score of 0.5; if boundary 2 = −0.797 is obtained, it indicates a 50% probability of achieving a score of 1; while a boundary 3 = 4.563 indicates a 50% probability of achieving a score of 2. The category response curves (CRCs) for all the items of the ROCF copy can be seen in Supplemental Material Figure S1.

Regarding the ROCF immediate recall, CFA provides evidence of the unidimensionality (χ^2^/df = 2.010; CFI = 0.985; TLI = 0.983; RMSEA = 0.050 [0.042; 0.059]; SRMR = 0.069). The ROCF immediate recall ordinal’s alpha was α = 0.92 and the McDonald’s omega was ɷ statistics = 0.89. Likewise, the discrimination of the items was between 0.873 (item 18: square and diagonal) and 2.037 (item 2: large rectangle). Like the ROCF immediate recall, the boundary 1 parameters indicate the value of the latent trait that yields a probability of 50% of obtaining a score of 0.5 versus 1, 1.5, or 2. Moreover, the CRCs for items 1 to 6 of the ROCF immediate recall suggested a low ability to reach the highest scores, unlike items 8 to 10, which require a higher level of skill to respond correctly (see Table 1). The remaining items demonstrated a better balance. To see the response category curves for all the items, please refer to Supplementary Materials Figure S2.

#### 3.1.2. Shortened Version of the Token Test

For the SVTT, CFA indicates the presence of unidimensionality (χ^2^/df = 1.171; CFI = 0.991; TLI = 0.991; RMSEA = 0.021 [0.013; 0.027]; SRMR = 0.144). The SVTT obtained an ordinal alpha of α = 0.96, and a McDonald’s omega ɷ of 0.89.

The item analysis based on IRT (see Table 2) reveals a progression in difficulty, with the initial items being considerably easier, such as “Touch a black one” (*b* = −4.10) and “Touch a red one” (*b* = −3.98). In contrast, the more complex items exhibit higher *b* values, indicating greater difficulty, such as “Put the red circle between the yellow square and the green square” (*b* = −0.15) and “In addition to touching the yellow circle, touch the black circle” (*b* = −0.26). In terms of discrimination, some items stand out for their high ability to differentiate between skill levels, such as “Touch a white one” (*a* = 2.27) and “Touch a green one” (*a* = 2.24), while others show lower discrimination values, suggesting a reduced capacity to distinguish between participants, such as “If there is a blue circle, touch the red square” (*a* = 0.89). The item characteristic curves (ICCs) for the SVTT can be seen in Supplemental Material Figure S3.

#### 3.1.3. Peabody Picture Vocabulary Test-III

For the PPVT-III, CFA provides evidence of unidimensionality in the factorial structure (χ^2^/df = 2.484; CFI = 0.953; TLI = 0.952; RMSEA = 0.061 [0.06; 0.062]; SRMR = 0.187). The ordinal alpha was α = 0.99 and the McDonald’s omega was ɷ = 0.98.

Due to the absence of variability in the data, as all the subjects answered correctly, items 2, 4, 5, 10, 11, and 20 were excluded from the IRT analysis. The discrimination values ranged from 0.45 (item 12 [climb]) to 26.489 (items 184 [melancholic], 185 [twins], 186 [plough], 187 [conflagration], 191 [terrace], and 192 [osculum]). The difficulty of the items ranged from −13.43 (item 12 [climb]) to 2.49 (items 167 [to inundate] and 181 [frieze]). The item response parameters and the ICCs for the PPVT-III can be seen in Table S1 and Figure S4 of the Supplementary Materials.

### 3.2. Demographic Variables’ Effect on Neuropsychological Performance

#### 3.2.1. ROCF Copy and Immediate Recall

The final models showed no multicollinearity (maximum VIF value was 3.94). Also, the maximum Cook’s distance was 0.43, indicating there were no influential values on the models. The results of the Levene test showed that the model satisfied the assumption of homoscedasticity for the ROCF copy (*p*-value = 0.90) and ROCF immediate recall (*p*-value = 0.30). To evaluate the normality, the Kolmogorov–Smirnov test was applied, yielding a normal distribution for the ROCF copy (*p*-value = 0.44) and ROCF immediate recall (*p* value = 0.80). The final multiple linear regression models for the theta scores of the ROCF copy and ROCF immediate recall were significant compared with the null model (*p*’s < 0.001).

**Figure 1:**
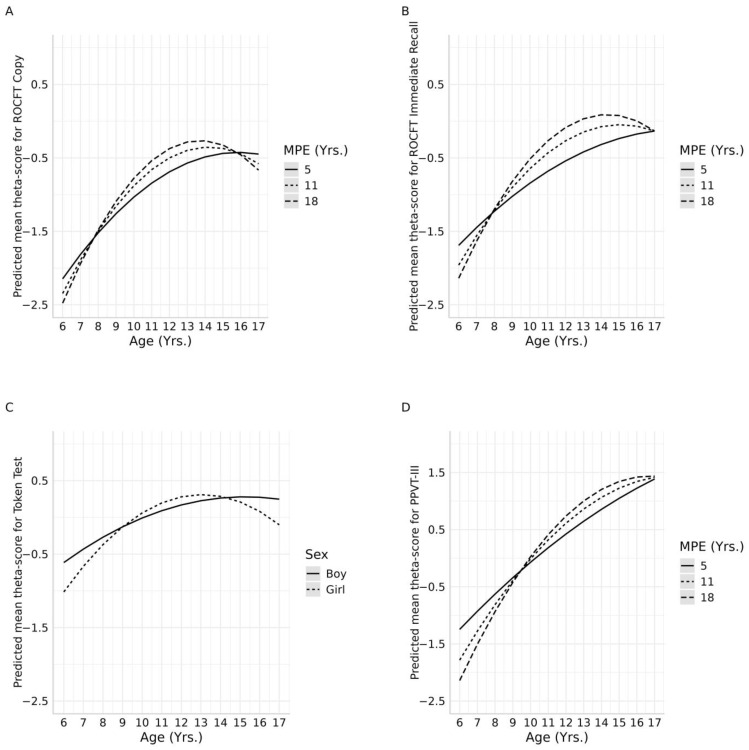
Mean predicted ability scores for each regression model. Subfigures (**A**–**D**) show the interactions between age and MPE or sex.

The ROCF copy ability score was influenced by the interactions Age^2^∗*In*(MPE), which shows that from 7 to 8 years old, the performance is better in children with lower MPE, but from 8 to 16 years old, their performance is the lowest compared to their peers with higher MPE. At the age of 16, the performance equalizes regardless of the MPE (see Table 3 and Figure 1A). The model explained 35% (*R*^2^ = 0.35) of the variance in the score and the overfitting percentage was 1.5%.

The ROCF immediate recall was affected by the interaction between Age^2^∗*In*(MPE), in which 8-year-old boys with parents having a mean of 5 years of education start achieving better results in the test, lowering their performance after turning 9 years old until 15 years old. In this age range, children and adolescents whose parents had a mean of 11 and 7.89 years of education obtained better scores. However, after turning 16 years old, they showed a similar performance regardless of the MPE (see Figure 1B). This implied the presence of a curvilinear relationship between the variables, positive for the first interaction and negative for the second one. The model explained 25% (*R*^2^ = 0.25) of the variance in the ability score and the overfitting percentage was 1.2%.

#### 3.2.2. Shortened Version of the Token Test

This test showed no evidence of multicollinearity (maximum VIF = 1.49) and the maximum Cook’s distance was 0.05. The Levene test showed consistent variance throughout the entire measurement (*p*-value = 0.02). Additionally, the normality of the residuals was demonstrated when applying the Kolmogorov–Smirnov test (*p*-value = 0.85). The final multiple linear regression model for the SVTT was significant (*p*-value < 0.001). The SVTT performance was affected by the interaction effect of Age^2^∗Sex, with a positive relation between the variables and a higher score achievement in 7- to 9- and 14- to 17-year-old male children and adolescents, while girls showed better performance during the age range of 9 to 14 years old (see Figure 1C). The model explained 12.5% (*R*^2^ = 0.125) of the variance in the score and the overfitting percentage was 1.03%.

#### 3.2.3. Peabody Picture Vocabulary Test-III

For the PPVT-III, there was no evidence of multicollinearity (maximum VIF = 3.94), and the maximum Cook’s distance was 0.11. The normality of the residuals was demonstrated with the Kolmogorov–Smirnov test (*p*-value = 0.505). The final multiple linear regression model for the PPVT-III was significant (*p*-value < 0.001). Performance on the PPVT-III was influenced by the interaction effect of Age^2^∗*In*(MPE). Children with lower MPE achieved higher scores than children with higher MPE until 8 years old, but after the age of 9 to 17, children whose parents had higher years of education performed better (see Figure 1D). The model explained 50% (*R*^2^ = 0.50) of the variance in the score and the overfitting percentage was 1.07%.

### 3.3. Normative Data Application

An online calculator based on the platform https://www.rstudio.com/products/shiny/ was created. This will facilitate probability calculation as clinicians should only include individual information requested in the calculator (i.e., scores for the specific test, age, MPE, and sex). The calculator transforms the item-by-item scores into ability scores, which are then adjusted according to the sociodemographic variables following the steps outlined in Section 2.4. Consequently, the clinician will obtain the z-score and percentile corresponding to their patient. This tool is freely available for all users at https://elianafuentes.shinyapps.io/Datos-Normativos/ (accessible from 15 February 2025).

## 4. Discussion

This study aimed (1) to describe the psychometric properties of the SVTT, the ROCF, and the PPVT-III through the IRT approach, and (2) to establish regression-based normative data for Waranka children and adolescents based on their IRT ability scores. What follows is a discussion of this study divided by the type of analysis and instruments.

### 4.1. Psychometric Properties

#### 4.1.1. ROCF Copy and Immediate Recall

For the ROCF copy and ROCF immediate recall, CFA demonstrated unidimensionality [37], which aligns with the results of [38], who identified a consistent and moderate to strong relationship between the responses to the test and the underlying dimension, implying that the unidimensional structure effectively captures the observed patterns in the scores for both the ROFC copy and immediate recall. Additionally, both the ROCF copy and immediate recall demonstrated adequate reliability through the Cronbach’s alpha and omega coefficients. The ROCF reliability has also been previously demonstrated, albeit through different methods [39,40,41].

Regarding the parameters generated through the IRT analyses for both the ROCF copy and the ROCF immediate recall, this study identified variability in the discrimination values, indicating that certain items were more effective in distinguishing abilities within the sample than others. For example, in the ROCF copy, items such as “Circle with three dots” (a = 2.59) and “Large rectangle” (*a* = 2.03) demonstrated excellent discrimination, suggesting that they effectively differentiated individuals across varying ability levels. Conversely, items like “Small segment” (*a* = 1.32) and “Square” (*a* = 0.87) exhibited lower discrimination, indicating a reduced capacity to distinguish between participants with differing abilities. However, most of the items demonstrated either excellent (*a* ≥ 2.00) or good discrimination scores (1.00 ≤ a < 2.00), with a small minority showing moderate (0.50 ≤ a <1.00) discrimination levels. Based on these findings, we do not recommend outright elimination of the lower-performing items, as they remain in the adequate ranges and may still provide complementary information and contribute to the overall reliability of the test. Overall, the results underscore the importance of considering item-level data to optimize the test performance. Items with high discrimination should be prioritized for interpretation, while low-performing items may require further review.

These findings suggest that the ROCF copy task is well suited for use in populations with a broad range of abilities, including younger children or individuals with cognitive impairments. In clinical settings, it can serve as an accessible tool to screen for visual–spatial deficits. However, clinicians should interpret the scores cautiously in high-functioning individuals, as the task may lack sufficient difficulty to challenge them. Conversely, the ROCF immediate recall task is better suited for evaluating higher-order memory processes, particularly in populations with moderate to high cognitive abilities. This task could be prioritized when detailed insights into memory and recall abilities are required. Future studies or adaptations of these tasks may consider refining the difficulty of items in the immediate recall task to ensure better coverage of lower ability ranges.

#### 4.1.2. Shortened Version of the Token Test

For the SVTT, the results of the CFA demonstrated unidimensionality. Additionally, the SVTT exhibited strong reliability, as evidenced by the ordinal alpha and McDonald’s omega. Although there is scant research on the psychometric properties of the SVTT, psychometric studies of other versions of the Token Test have demonstrated adequate reliability based on the ordinal alpha [42,43].

The parameters of this test generated through IRT indicated a range of discrimination values, with some items demonstrating strong differentiation between individuals of different ability levels, while others were less effective. For example, items such as “Touch the green one” (*a* = 2.24) and “Touch the white one” (*a* = 2.27) exhibited high discrimination, indicating their effectiveness in distinguishing between participants with varying skill levels. In contrast, items like “If there is a blue circle, touch the red square” (*a* = 0.89) and “Touch the red circle—no—the white square” (*a* = 0.99) showed lower discrimination, suggesting they were less effective in differentiating abilities. Most items showed either excellent (*a* ≥ 2.00) or good discrimination values (1.00 ≤ *a* < 2.00), with a small minority showing moderate (0.50 ≤ *a* < 1.00) discrimination levels.

The wide range of item difficulty values observed illustrates variability that enhances the test’s ability to measure a broad spectrum of abilities. For instance, items such as “Touch a black one” (*b* = −4.10) and “Touch a red one” (*b* = −3.98) reflect low difficulty, indicating that a minimal level of skill is sufficient to answer correctly. Conversely, items like “In addition to touching the yellow circle, touch the black circle” (*b* = −0.26) and “Put the red circle next to the yellow square” (*b* = −0.15) represent higher difficulty, requiring greater ability to achieve correct responses. This range of difficulty values ensures that the test captures the performance across varying ability levels, although the ICC analysis suggests that many early items may be too easy for higher-skilled participants. Such items may be excessively easy and may not effectively distinguish among individuals with higher abilities. This is consistent with the test’s original design, which was intended for populations with aphasia, where easier items were necessary for accessibility. Considering this, we do not recommend revising or replacing the items with low discrimination and greater ease, as they serve an important purpose with clinical populations. Furthermore, this supports the claims of test validity and internal consistency made in other sections of this paper. However, future studies may consider altering items so that the test may be applicable to those with a broader range of cognitive–linguistic abilities, and/or consider the differential performance of clinical vs. non-clinical groups.

#### 4.1.3. Peabody Picture Vocabulary Test-III

The CFA results indicated the presence of unidimensionality in the PPVT-III. Regarding reliability, the PPVT-III showed strong internal consistency through the ordinal alpha and McDonald’s omega coefficients. The high values obtained for both coefficients suggest that the test is reliable for assessing vocabulary skills in this population.

Analysis of the discrimination parameters indicated wide variability among the test items, again showing that some items are more effective in distinguishing between individuals’ ability levels. Six items were excluded from the IRT analysis due to the lack of variability in the data, as all the subjects answered correctly concerning these items. Given that these items represent only a small fraction (2.1%) of the total pool of 192 items, their exclusion is unlikely to have a significant effect on the estimation of item parameters or person abilities.

Regarding the difficulty parameters, the variability observed indicates that the test effectively spans a range of ability levels, capturing both easier and more challenging cognitive tasks. Importantly, the arrangement of the test items reflects an age-appropriate progression of difficulty, suggesting that the instrument is well calibrated for the developmental characteristics of the Waranka children population. This alignment supports the test’s validity in measuring cognitive constructs across a wide ability spectrum, making it suitable for both younger and older children within the assessed age range.

Finally, according to the CRC for the PPVT-III, particularly for the first 40 items, individuals with a low level of ability were able to attain the highest scores, indicating that these initial items were relatively easier and required minimal proficiency. This pattern could partially be explained by the inclusion criteria used in this study, which required participants to meet a minimum threshold of cognitive ability (IQ ≥ 80). Consequently, the sample may have been skewed toward individuals capable of easily completing these simpler items, limiting the ability of these items to differentiate among participants. Furthermore, the structured progression of difficulty in the PPVT-III means that the earlier items are intentionally designed to be accessible to a wide range of respondents, including those with lower ability levels.

Fatigue may also have influenced the results, as participants might have experienced diminished engagement or focus during the latter stages of the test. However, this seems less likely to explain the observed pattern, as these easier items appeared early in the test and required minimal effort. Additionally, the high reliability coefficients obtained for the PPVT-III suggest that the effect of fatigue, if present, did not significantly impact the test’s internal consistency.

### 4.2. Demographic Variables and Normative Data

The main objective of this study was to obtain normative data for the Waranka population using a regression-based approach. The effects of demographic variables on the test performance were analyzed using multiple linear regression based on the ability (*θ*) and including as predictors Age, Age^2^, *In*(MPE), sex, and all the possible two-way interactions. Orthogonality (Age^2^) ensures that covariates are independent of one another (the independence assumption between covariates). This approach offers several advantages. First, it eliminates multicollinearity, allowing for the precise estimation of each term’s unique contribution to the outcome. Second, it provides clearer interpretation by ensuring that the effects of individual terms, such as the linear and quadratic age components, can be understood separately. Third, it reduces bias by preventing additional terms in the model from distorting the estimates of other covariates. Finally, orthogonality enhances the robustness of the model, making it more stable and less susceptible to variability in the data [44]. The natural logarithmic scale *In*(MPE) was used to model the relationship between parents’ years of education and test performance, capturing the expected rapid initial improvement in performance during the early years of education, followed by a gradual deceleration as additional years of schooling yielded diminishing returns.

The sociodemographic variables were tested for their influence on performance, individually and interacting with each other. This allowed us to capture in a more nuanced way the influence of sociodemographic variables on children’s test scores and, thus, to ensure that the normative data adequately represent the characteristics of the assessed population. In addition, explaining the relationship between these variables helps to understand their cognitive performance and the normative data that are supposed to capture it.

#### 4.2.1. ROCF Copy and Immediate Recall

The results of the multiple linear regression models for the ROCF copy indicates that the interaction of Age^2^ and MPE influenced performance in our study. At the age of 7, children whose parents have an average of 5 years of education perform better initially, but their scores decrease in the 8- to 16-year-old range. However, children of the same age range whose parents have an average of 11 and 18 years of education increase their performance on the test. The different performance levels in children of the same age but with parents of different educational levels highlight the important role of the parent’s education in youths’ cognitive development [45]. Moreover, as mentioned, previous results indicate that better performance in the Spanish-speaking pediatric population is associated with a higher parental educational level [46].

Regarding the ROCF immediate recall scores, the Age^2^ and MPE interaction had an effect on performance. For parents with 4 years of education, their 8-year-old children have good performance in the test, but once they reach 9 to 15 years of education, their scores decrease. On the contrary, children in the same age range whose parents have an average of 8 and 11 years of education have better performance. From the age of 16, the performance seems to equalize, being similar in adolescents regardless of their parents’ years of education. This suggests that the number of years of education parents have plays an important role in youths’ performance between the ages of 9 and 15. However, from 16 years onwards, other factors may come into play, leading to similar performance in adolescents regardless of parental education. This is consistent with results from Cermakova et al. [45], which indicate that parental education plays a significant role in the overall cognitive performance in childhood, but this influence decreases as children mature.

#### 4.2.2. Shortened Version of the Token Test

The results of the analyses suggest that the statistical model used for the SVTT was reliable and robust. The interaction effect between Age^2^ and sex was significant, implying a combined effect of these variables on the test performance. Overall, the model accounted for 13% of the variability in test scores, emphasizing the role of age and sex in predicting performance on the SVTT. The results indicate higher scores in boys 7 to 9 years old and 14 to 17 years old, while girls perform better than boys at the age range of 9 to 14 years old. This association differs from the outcomes of previous studies, which have found no association between gender and score in Spanish speaking children [47], young adults [48], and older adults [49]. This lack of association has also been found for the Revised Version of the Token Test in Mexican children [43].

#### 4.2.3. Peabody Picture Vocabulary Test-III

The multiple lineal regression model applied to the PPVT-III data demonstrated robustness. The model was significant and explained 53% of the variance in the score. Performance was significantly influenced by the interaction between Age^2^ and MPE, in which 8-year-old children whose parents have an average of 4 years of education perform better than those who turn 9 years old, the time at which performance decreases until the age of 17. In the same age range (9 to 17), higher scores are achieved by children whose parents have an average of 11 and 8 years of education. This coincides with previous results indicating an association between the MPE and the PPVT scores in the Spanish-speaking pediatric population [50]. Moreover, the literature supports the role of MPE in vocabulary development, showing better performance on verbal fluency tests among children with parents who have a higher level of education in comparison with children with parents with lower education [51].

### 4.3. Strengths, Limitations and Clinical Implications

There are few recent studies dedicated to establishing normative data for the ROCF, the SVTT, and the PPVT-III in Spanish-speaking children, especially in Latin America. Although there are previous normative data studies in an Ecuadorian population, to our knowledge, this is the first study to obtain normative data in children belonging to an under-represented ethnic population in Ecuador, the Waranka. Our study’s outcomes indicate that the tests administered are valid for the assessment of Waranka children. Moreover, our results provide normative data for that purpose in that specific population. Furthermore, few studies have aimed to evaluate the psychometric properties and norming of these tests through IRT models. This represents an important strength due to the superiority of this approach over classical test theory, given its several advantages [17].

The above has important clinical implications. Firstly, the psychometric properties obtained in the present study allow for consideration of various factors when applying the tests and thus offer more accurate clinical and research outcomes. Secondly, having normative data for Waranka children enables precise clinical outcome assessments utilizing the tests included in this study, allowing for accurate evaluation of children’s skills and establishing a better standard for identifying difficulties in terms of those skills. This, in turn, facilitates more accurate detection of certain clinical diagnoses. In addition, the test norms provided in this study are useful for educational purposes, with language being one of the most important areas assessed in schools through psychopedagogical evaluations.

This study has several limitations that should be considered. First, our sample was drawn exclusively from the Waranka population in Ecuador, so the findings may not be directly generalizable to other indigenous groups or under-represented communities in Ecuador and Latin America. Differences in cultural practices, linguistic norms, and socioeconomic conditions can substantially influence performance on these tests. Second, although many participants may have been bilingual in Spanish and Kichwa (or another indigenous language), we did not formally assess or control for bilingualism. This is especially relevant for vocabulary-based measures such as the PPVT-III, where different levels of Spanish fluency could affect comprehension and word retrieval. Third, we did not collect detailed information on the participants’ cultural contexts (e.g., degree of adherence to traditional practices, language use at home), which could also shape test performance. Future research should incorporate standardized evaluations of bilingualism, include a broader range of under-represented groups, and explicitly assess cultural variables to enhance the validity and comparability of neuropsychological test norms across diverse populations. Finally, four items (2, 4, 5, and 10) were excluded from the IRT analysis of the PPVT due to the lack of variability, as all the participants answered them correctly. Although their exclusion is unlikely to have a significant effect on the estimation of the item parameters or person abilities, we acknowledge that the absence of variability in some items may reflect a ceiling effect in certain sections of the test.

## 5. Conclusions

This is the first study to both analyze the psychometric properties of the ROCF, SVTT and PVT-III through IRT models and generate normative data in an under-represented first nation in Ecuador. Counting with normative data in the Waranka children population is relevant for clinical and research purposes, allowing for comparison of children’s abilities with the normative data obtained in this study, thus enabling the detection of potential deficits to be properly addressed. Moreover, IRT models enable us to have psychometric properties of the test that are accurate and, therefore, relevant for the validity of the obtained data.

## Figures and Tables

**Table 1:** ROCF item parameters based on item responses theory.

Item	ROCF Copy				ROCF Immediate Recall			
	Boundary 1	Boundary 2	Boundary 3	*a*	Boundary 1	Boundary 2	Boundary 3	*a*
Item 1 [cross]	−2.168	−0.797	4.563	2.11	−0.931	1.18	5.102	1.089
Item 2 [large rectangle]	−1.614	−0.834	7.093	2.404	−1.143	−0.209	3.474	2.037
Item 3 [diagonal cross]	−1.419	−0.578	10.19	2.036	−0.428	0.285	4.044	1.595
Item 4 [horizontal median]	−1.672	−0.917	6.629	2.192	−0.845	−0.204	4.01	1.698
Item 5 [vertical median]	−1.931	−1.081	5.839	1.974	−0.759	−0.132	3.768	1.704
Item 6 [small rectangle]	−1.952	−0.775	6.204	2.033	−0.633	1.086	6.558	0.911
Item 7 [small segment]	−1.059	−0.618	18.061	1.329	1.192	1.675	4.402	1.241
Item 8 [four parallel lines]	−1.475	−0.237	5.069	1.899	0.239	1.575	4.658	1.173
Item 9 [triangle]	−1.737	−0.834	8.723	1.843	0.198	1.179	5.599	1.048
Item 10 [small perpendicular]	−1.66	−0.839	7.871	1.852	2.187	3.067	4.811	1.06
Item 11 [circle with 3 dots]	−2.08	−0.975	4.29	2.598	−1.144	0.414	4.24	1.514
Item 12 [five parallel lines]	−1.999	−0.808	6.058	1.946	−0.085	1.042	5.346	1.121
Item 13 [isosceles]	−2.017	−0.862	4.304	2.517	−1.4	0.094	4.335	1.692
Item 14 [rhombus]	−1.793	−0.566	8.214	1.684	−1.039	0.444	5.277	1.092
Item 15 [vertical segment]	−2.068	−0.986	5.636	1.901	−0.259	0.937	6.259	0.914
Item 16 [extension right]	−1.921	−0.943	5.367	2.091	−0.625	0.27	5.15	1.066
Item 17 [lower cross]	−1.903	−0.873	6.251	2.193	−0.603	1.262	5.426	1.062
Item 18 [square and diagonal]	−2.413	−1.209	5.604	2.182	−1.268	1	6.544	0.873

Note. 1 Boundary = response category 0.5; 2 Boundary = response category 1; 3 Boundary = response category 2. *a* = Discrimination.

**Table 2:** Shortened Version of the Token Test item parameters, based on item response theory.

Items	*a*	*b*	Items	*a*	*b*
#01 [Touch a circle]	1.06	−3.97	#19 [Touch the white circle and the red circle]	1.39	−1.02
#02 [Touch a square]	1.29	−3.89	#20 [Touch the large ·white circle and the small green square]	1.57	−0.89
#03 [Touch a yellow token]	1.41	−3.26	#21 [Touch the small black circle and the large yellow square]	1.57	−1.20
#04 [Touch a red one]	1.42	−3.98	#22 [Touch the large green square and the large red square]	1.90	−1.32
#05 [Touch a black one]	1.35	−4.10	#23 [Touch the large white square and the small green circle]	1.56	−1.40
#06 [Touch a green one]	2.24	−2.93	#24 [Put the red circle on the green square]	1.97	−0.87
#07 [Touch a white one]	2.27	−3.16	#25 [Touch the black circle with the red square]	1.37	−0.30
#08 [Touch the yellow square]	1.42	−2.43	#26 [Touch the black circle and the red square]	1.19	−1.14
#09 [Touch the black circle]	1.65	−2.62	#27 [Touch the black circle or the red square]	1.42	−0.70
#10 [Touch the green circle]	1.47	−2.38	#28 [Put the green square away from the yellow square]	1.13	−1.23
#11 [Touch the white square]	1.47	−2.56	#29 [If there is a blue circle, touch the red square]	0.89	−0.72
#12 [Touch the small white circle]	1.27	−2.28	#30 [Put the green square next to the red circle]	1.28	−0.51
#13 [Touch the large yellow square]	1.11	−2.47	#31 [Touch· the squares slowly and the circles quickly]	2.05	−0.79
#14 Touch the large green square]	1.06	−3.49	#32 [Put the red circle between the yellow square and the green square]	1.46	−0.15
#15 Touch the small black circle]	1.18	−2.56	#33 [Touch all the circles, except the green one]	1.99	−1.18
#16 [Touch the red circle and the green square]	1.39	−1.29	#34 [Touch the red circle—no—the white square]	0.99	−1.36
#17 [Touch the yellow square and the black square]	1.27	−1.35	#35 [Instead of the white square, touch the yellow circle]	1.82	−1.32
#18 [Touch the white square and the green circle]	2.02	−1.55	#36 [In addition to touching the yellow circle, touch the black circle]	1.09	−0.26

Note. *a* = Discrimination parameters; *b* = Difficulty parameters. This symbol “#” indicates the item number.

**Table 3:** Final multiple linear regression models.

Score	Predictor	Estimate	Std. Error	*t*	Sig.	Adjusted *R*^2^	*SD_e_*
ROCF copy	Intercept	−1.121	0.164	−6.840	<0.001	0.347	0.758
	Age	8.945	3.037	2.945	0.003		
	Age^2^	2.657	2.996	0.887	0.375		
	*In*(MPE)	0.102	0.075	1.346	0.178		
	Age*In(MPE)	0.392	1.421	0.276	0.782		
	Age^2^*In(MPE)	−3.561	1.394	−2.554	0.011		
ROCF immediate recall	Intercept	−1.046	0.190	−5.486	<0.001	0.253	0.882
	Age	5.226	3.534	1.479	0.140		
	Age^2^	5.064	3.485	1.453	0.147		
	*In*(MPE)	0.188	0.088	2.139	0.033		
	Age*In(MPE)	2.053	1.653	1.242	0.214		
	Age^2^*In(MPE)	−4.002	1.622	−2.467	0.014		
SVTT	Intercept	0.028	0.064	0.437	0.662	0.125	0.855
	Age	4.886	1.286	3.797	<0.001		
	Age^2^	−2.197	1.278	−1.719	0.086		
	Sex (Girl)	−0.051	0.086	−0.593	0.553		
	Age*Sex (Girl)	0.129	1.726	0.075	0.940		
	Age^2^*Sex (Girl)	−3.385	1.721	−1.967	0.049		
PPVT-III	Intercept	0.162	0.182	0.886	0.376	0.50	0.84
	Age	7.031	3.388	2.075	0.038		
	Age^2^	5.945	3.143	1.779	0.075		
	*In*(MPE)	0.038	3.341	0.456	0.648		
	Age*In(MPE)	4.536	0.084	2.862	0.004		
	Age^2^*In(MPE)	−4.230	1.584	−2.720	0.006		

Note: ROCF = Rey–Osterrieth Complex Figure; MPE = mean parents’ years of education; SVTT = Shortened Version of the Token Test; PPVT-III = Peabody Picture Vocabulary Test-III. The asterisk (*) represents a statistical interaction. The superscript “^2^” denotes a quadratic effect.

## Data Availability

The data and code can be provided upon request.

## Supplementary Materials

The following supporting information can be downloaded at https://www.mdpi.com/article/10.3390/healthcare13040423/s1. In this link it can be downloaded Table S1: Peabody Picture Vocabulary Test-III Item Response Parameters, based in Item Response Theory, Figure S1: ROCF-Copy Category response curves, Figure S2 ROCF-Immediate recall Category response curves, Figure S3: Shortened Version Token Test Item Characteristic Curves, and Figure S4: Peabody Picture Vocabulary Test-III Test Item Characteristic Curves. The R script with data analyses is available in https://github.com/diegoriveraps/irt_waranka (accessible from 15 February 2025).
