# Supplementary material for: Psychometric Properties and Normative Data Using Item Response Theory Approach for Three Neuropsychological Tests in Waranka Children Population
Source: Healthcare (Basel). 2025 Feb 15;13(4):423. doi: 10.3390/healthcare13040423 (PMC11855604; doi:10.3390/healthcare13040423)

Supplemental Material

Table S1 Peabody Picture Vocabulary Test-III Item Response Parameters, based in Item Response Theory.

Item	*a*	*b*	Item	*a*	*b*	Item	*a*	*b*
#1: Broom	8.672	-3.486	# 69: Cliff	1.08	-0.775	# 131: Links	2.642	1.563
#3: Plane	1.856	-4.552	# 70: Terror	0.776	-2.249	# 132: Fictitious	2.817	1.149
# 6: Lamp	1.267	-5.041	# 71: to direct	1.426	-1.678	# 133: Spherical	5.899	1.322
# 7: Money	5.918	-3.558	# 72: Walrus	0.923	-0.791	# 134: Primate	3.962	1.653
# 8: Helicopter	2.902	-3.643	# 73: Palm	1.244	-0.635	# 135: Calm	3.528	1.654
# 9: Fence	1.743	-3.076	# 74: Predator	1.096	-0.935	# 136: to replace	3.299	1.956
# 12: Climb	0.45	-13.434	# 75: Funnel	1.365	-1.266	# 137: Peninsula	3.495	1.92
# 13: Cow	2.86	-3.942	# 76: to refuel	0.722	0.496	# 138: Perpendicular	2.789	1.948
# 14: to swim	1.089	-5.186	# 77: Adjustable	0.966	-1.355	# 139: Newspaper	3.002	1.742
# 15: Empty	2.12	-3.562	# 78: Rodent	1.065	0.014	# 140: Obelisk	2.916	1.816
# 16: to dig	1.234	-4.336	# 79: to crash	0.8	0.304	# 141: to ponder	2.854	1.938
# 17: Farmer	3.152	-3.412	# 80: Thermos	1.055	-0.822	# 142: Incandescent	4.89	1.674
# 18: Accident	1.413	-4.726	# 81: Arctic	0.933	-1.064	# 143: Incisor	3.625	1.763
# 19: Nest	2.517	-3.761	# 82: to calculate	1.49	-0.604	# 144: Culinary	3.014	1.741
# 21: Envelope	1.514	-2.721	# 83: Triplets	1.044	-0.829	# 145: to steal	4.762	1.767
# 22: Castle	1.601	-3.257	# 84: to pollute	1.212	-0.973	# 146: Dromedary	4.494	2.137
# 23: to measure	1.44	-3.983	# 85: Bouquet	1.083	-0.136	# 147: to imprison	5.624	1.827
# 24: Kangaroo	1.101	-3.776	# 86: Waves	1.571	-0.083	# 148: Bovine	4.712	1.793
# 25: Fruit	1.1	-4.661	# 87: to leave	1.266	-0.654	# 149: Stamen	6.834	1.965
# 26: Chain	1.195	-3.68	# 88: Pod	1.17	0.265	# 150: Vestige	3.647	1.967
# 27: Cactus	1.006	-3.607	# 89: to classify	0.865	-0.009	# 151: Tutor	3.702	2.065
# 28: Porcupine	1.162	-3.495	# 90: Vine	1.099	0.951	# 152: to rub down	3.787	1.924
# 29: to yawn	1.239	-3.235	# 91: to dissect	1.202	0.443	# 153: Commercial	3.483	2.017
# 30: Goat	1.302	-3.091	# 92: Glider	1.031	1.312	# 154: Wildebeest	4.83	1.966
# 31: Decorated	1.658	-2.766	# 93: Succulent	1.209	0.559	# 155: Claw	5.332	1.976
# 32: Fox	1.156	-2.926	# 94: Pelican	1.282	0.681	# 156: horsewoman	4.136	1.944
# 33: Talons	1.2	-3.294	# 95: Yacht	1.178	0.182	# 157: to filter	9.752	1.959
# 34: to argue	1.407	-3.153	# 96: to welcome	1.126	0.151	# 158: Pentagon	5.595	2.093
# 35: Astronaut	1.477	-2.705	# 97: Archer	1.857	0.19	# 159: Foresight	8.022	1.961
# 36: to saw	1.323	-2.673	# 98: Mammal	1.926	0.497	# 160: Dock	6.774	2.166
# 37: trunk	0.802	-5.647	# 99: Composer	1.733	0.74	# 161: to converge	7.082	2.064
# 38: Huge	1.383	-3.399	# 100: Oasis	1.135	1.579	# 162: Receptacle	12.868	2.032
# 39: Parachute	1.355	-2.726	# 101: Citrus	1.661	0.361	# 163: Drilling	5.891	2.081
# 40: to deliver	1.224	-2.976	# 102: to lubricate	1.677	1.005	# 164: Vitreous	9.58	2.023
# 41: Globe	0.89	-4.366	# 103: Speedometer	1.661	0.793	# 165: To soar	9.308	1.906
# 42: Calculator	1.179	-1.915	# 104: Brew	1.573	0.976	# 166: Deciduous	3.796	2.287
# 43: to drip	1.12	-2.936	# 105: to raise	1.399	0.991	# 167: To inundate	3.739	2.491
# 44: Beehive	1.158	-1.987	# 106: Reprimand	1.947	0.602	# 168: Abrasive	8.832	2.114
# 45: to sand	1.379	-1.92	# 107: Porcelain	1.533	1.144	# 169: Palmiped	11.806	2.193

# 46: Statue	1.222	-2.477	# 108: Considerable	1.934	0.245	# 170: Shear	8.124	2.393
# 47: Terrified	1.204	-2.953	# 109: Railing	2.033	0.659	# 171: Marsupial	6.09	2.261
# 48: Rectangle	1.204	-2.608	# 110: Compass	2.233	0.722	# 172: Coniferous	7.022	2.304
# 49: Frame	0.984	-1.878	# 111: to instruct	1.985	1.076	# 173: Reckless	24.499	2.059
# 50: Baggage	1	-2.583	# 112: Devoid	1.629	1.106	# 174: Entomologist	26.465	2.099
# 51: handwriting	0.839	-3.176	# 113: Infinite	2.408	0.472	# 175: Baluster	11.801	2.193
# 52: to cheer	0.988	-1.896	# 114: Choreographic	2.389	1.006	# 176: Pecuniary	4.682	2.411
# 53: Vehicle	1.092	-3.183	# 115: Confidence	1.741	1.237	# 177: to inoculate	7.242	2.351
# 54: to polish	1.21	-1.638	# 116: Wedge	1.701	1.377	# 178: Embossed	24.705	2.058
# 55: Celery	1.729	-1.684	# 117: Equine	1.445	1.325	# 179: to dine	7.198	2.214
# 56: Oval	0.894	-2.426	# 118: Valve	1.732	0.959	# 180: Pachyderm	26.323	2.019
# 57: Vegetable	1.786	-0.56	# 119: to harvest	2.332	0.479	# 181: Frieze	6.078	2.493
# 58: Fluffy	1.044	-2.726	# 120: to moan	2.421	0.863	# 182: Caliper	11.755	2.323
# 59: to award	1.154	-2.712	# 121: Coil	2.929	1.026	# 183: Selenic	17.416	2.404
# 60: Brain	1.671	-2.442	# 122: Isolation	2.173	0.923	# 184: Melancholic	26.489	2.286
# 61: to bother	1	-2.42	# 123: Easel	2.63	1.166	# 185: Twins	26.489	2.286
# 62: File	1.015	-2.247	# 124: Reflection	2.023	1.722	# 186: to plough	26.489	2.286
# 63: Island	0.977	-1.728	# 125: Upholstery	2.372	1.667	# 187: Conflagration	26.489	2.286
# 64: to choose	1.13	-1.04	# 126: Device	2.819	1.175	# 188: to burden	11.755	2.323
# 65: Pair	1.403	-1.317	# 127: to groom	2.297	1.432	# 189: Nopal	17.456	2.404
# 66: Angle	1.108	-1.278	# 128: Erudite	2.355	1.783	# 190: to crop	17.456	2.404
# 67: Reptile	0.986	-0.714	# 129: Sedan	2.149	2.132	# 191: Terrace	26.489	2.286
# 68: Jaw	1.081	-0.76	# 130: Facade	2.571	1.685	# 192: Osculum	26.489	2.286

Note. a = Discrimination parameters; b = Difficulty parameters. This symbol “#” indicates the item number.

Figure S1 ROCF-Copy Category response curves

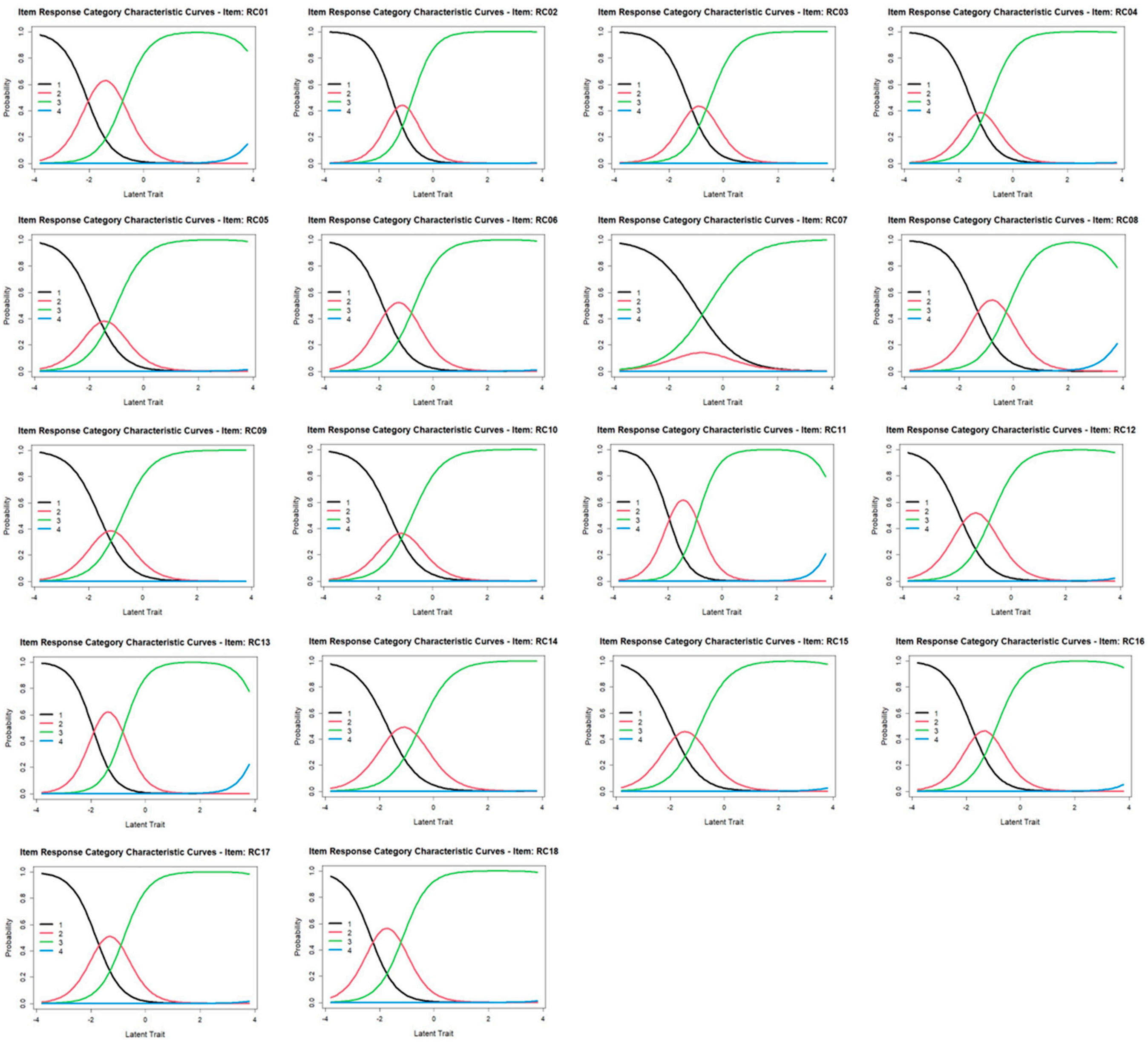

Note. Line 1= response category 2; Line 2= response category 1; line 3= response category = 0.5; line 4= response category = 0

Figure S2 ROCF-Immediate recall Category response curves

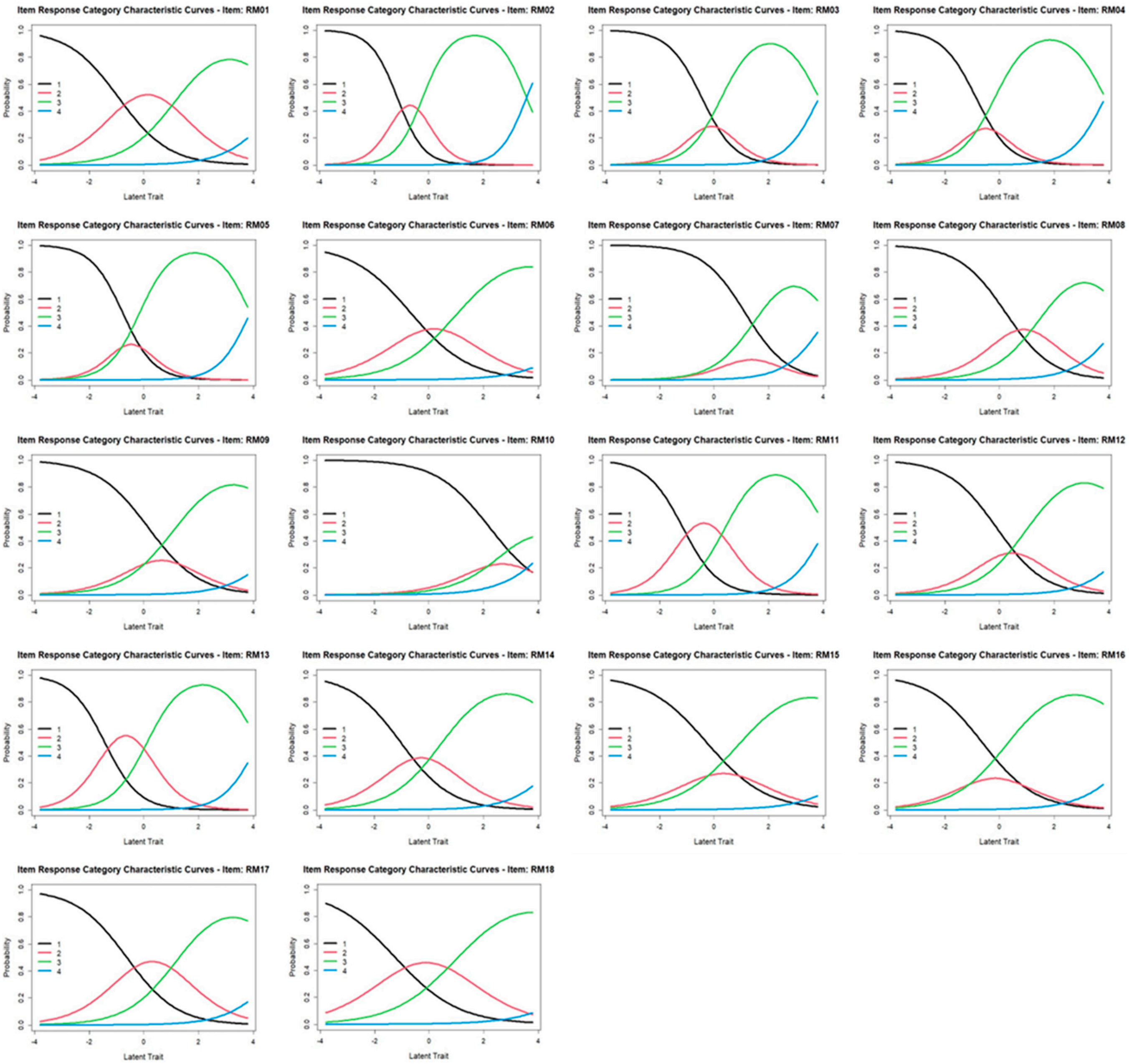

Note. Line 1= response category 2; Line 2= response category 1; line 3= response category = 0.5; line 4= response category = 0

Figure S3 Shortened Version Token Test Item Characteristic Curves

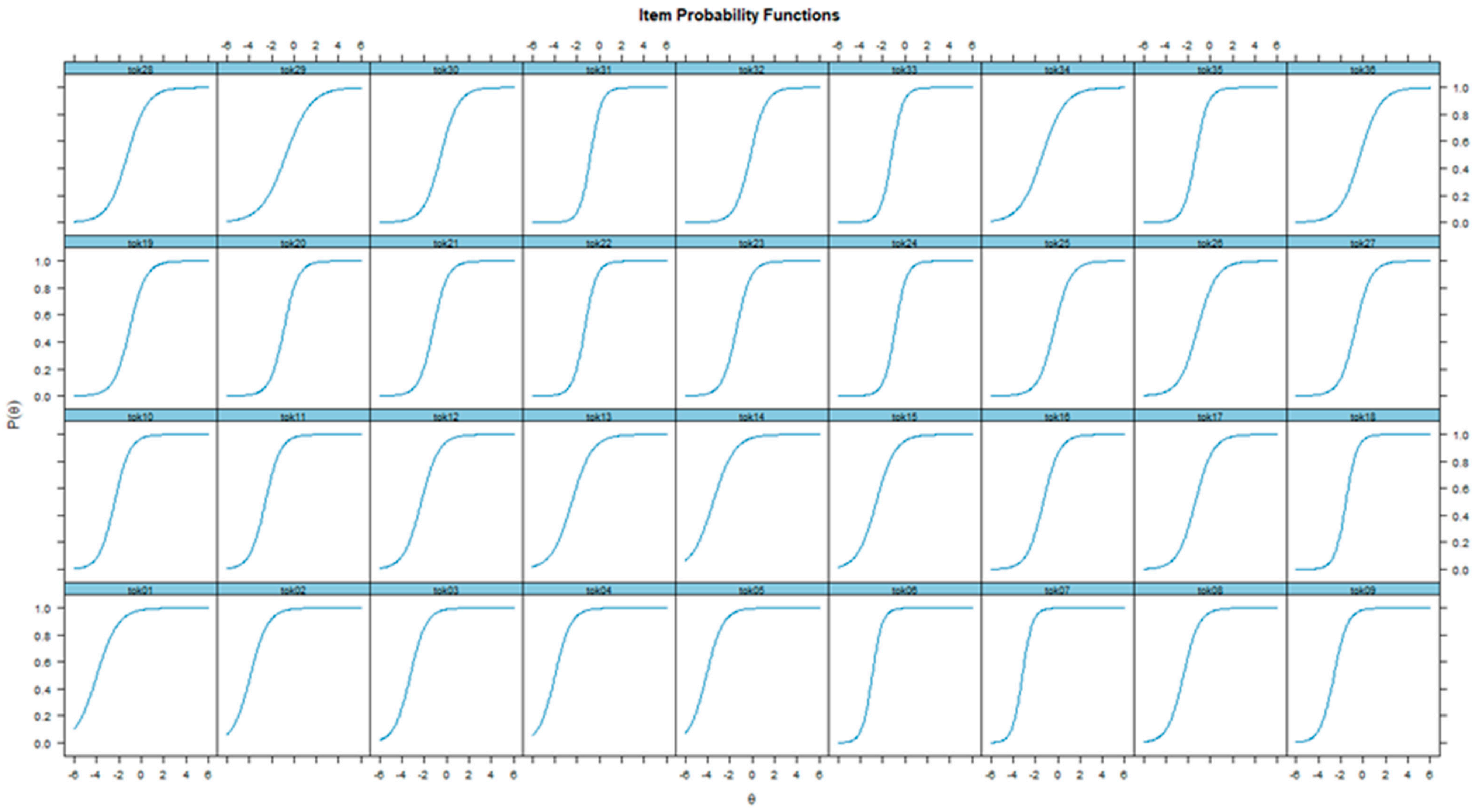

Figure S4 Peabody Picture Vocabulary Test-III Test Item Characteristic Curves

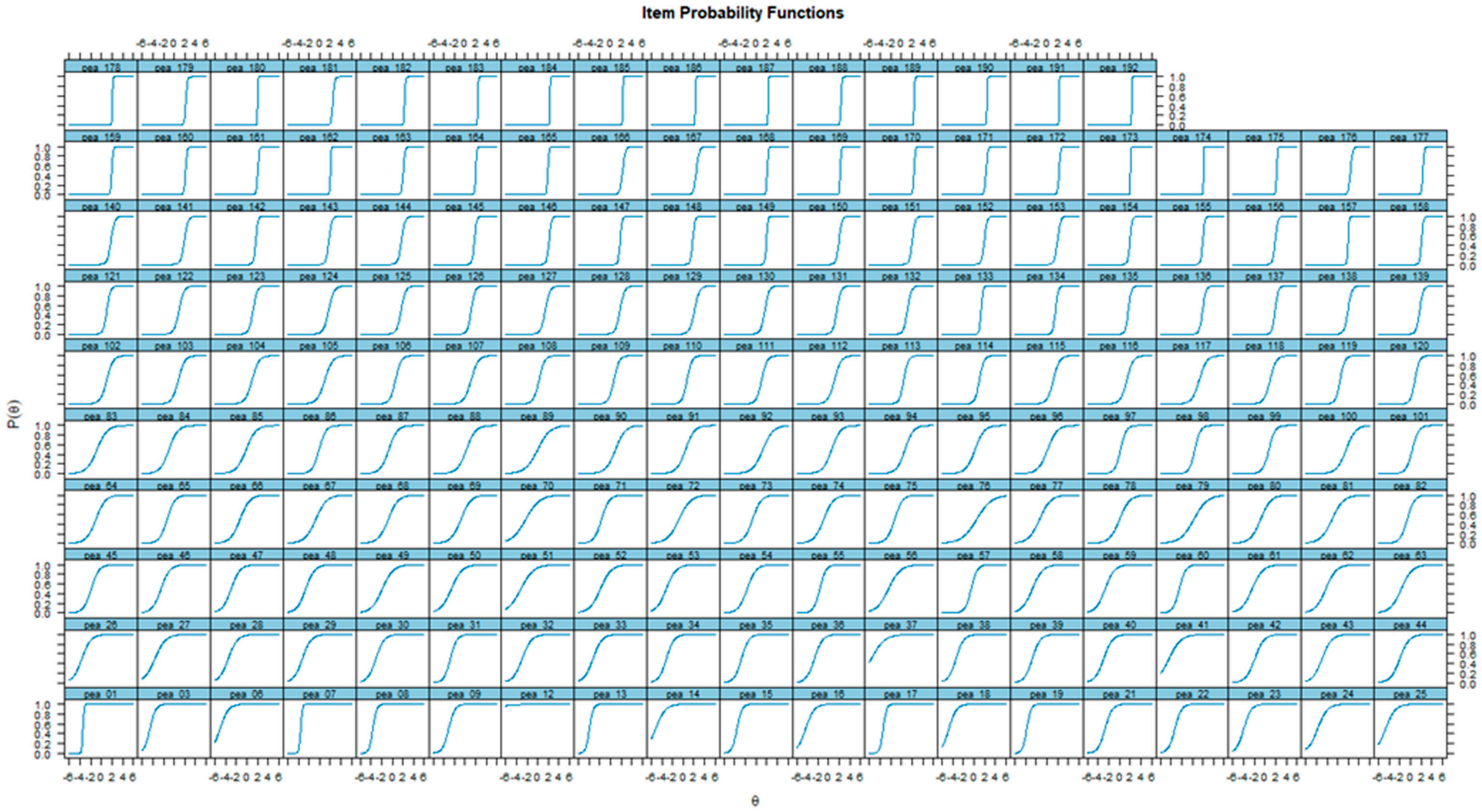

Supplement: Supplementary file 1 [file healthcare-13-00423-s001.zip › healthcare-3396822-supplementary.pdf]
